# State Lesbian, Gay, Bisexual, Transgender, and Queer (LGBTQ+) Policies and Subjective Cognitive Decline in US Sexual Minority Adults Aged 45 Years and Older

**DOI:** 10.1002/alz70860_096574

**Published:** 2025-12-23

**Authors:** Zhigang Xie

**Affiliations:** ^1^ University of North Florida, Jacksonville, FL, USA

## Abstract

**Background:**

In 2023, state legislatures across the United States introduced a record‐breaking 510 anti‐lesbian, gay, bisexual, transgender, and queer (LGBTQ+) bills. These anti‐LGBTQ+ bills may increase chronic stress and ultimately affect their overall health, including subjective cognitive decline (SCD).

**Method:**

Combining the 2019‐2022 Behavioral Risk Factor Surveillance System (BRFSS) and 2017‐2021 policy tallies, we conducted multivariable logistic regression analyses to explore the relationship between LGBTQ+ state policy environments (protective vs. restrictive) and SCD (yes vs. no) among sexual minority adults aged 45 years and older.

**Result:**

Among 10,487 sexual minority adults, 1,009 (9.9%) reported experiencing SCD. Participants from states with protective policies were less likely to report SCD than those from restrictive ones (adjusted odds ratio [AOR] = 0.63; 95% confidence interval [CI]: 0.46–0.88). Among those with SCD, individuals from protective states were more likely to discuss their cognitive issues with healthcare providers (AOR = 1.68; 95% CI: 1.04–2.72) and to receive the help they needed (AOR = 1.55; 95% CI: 1.05–2.29).

**Conclusion:**

Sexual minority adults experiencing SCD were more likely to reside in states with restrictive LGBTQ+ policies. Conversely, those with SCD living in states with protective policies were more likely to access cognitive‐related care and support.

